# Prevalence of HIV and hepatitis B virus among pregnant women in Luanda (Angola): geospatial distribution and its association with socio-demographic and clinical-obstetric determinants

**DOI:** 10.1186/s12985-021-01698-7

**Published:** 2021-12-04

**Authors:** Amélia Nkutxi Vueba, Ricardo Almendra, Paula Santana, Clarissa Faria, Maria do Céu Sousa

**Affiliations:** 1 Faculty of Pharmacy, University of Coimbra (FFUC), Coimbra, Portugal; 2 Centre of Studies on Geography and Spatial Planning (CEGOT), University of Coimbra, Coimbra, Portugal; 3 Centre of Studies on Geography and Spatial Planning (CEGOT), Department of Geography and Tourism, University of Coimbra, Coimbra, Portugal; 4 Center for Neuroscience and Cell Biology (CNC), University of Coimbra, Coimbra, Portugal

**Keywords:** Virus, Sexually transmission infections, Seroprevalence, HIV, Antibodies, HBsAg, HBeAg, ELFA, Socio-demographic characteristics, Kernel density function

## Abstract

**Background:**

HIV and HBV infections remain responsible for high rate of morbidity and mortality in many African Countries, affecting women and newborns. This study aims to analyze the spatial pattern of HIV and HBV infections in pregnant women in Luanda, Angola, and the statistical association between HIV and HBV and socio-economic characteristics, hygiene, and health status.

**Methods:**

Detection of anti-HIV antibodies (total anti-HIV-1, anti-HIV-2 and HIV-1 p24 antigen) and Hepatitis B antigens (HBsAg, HBeAg) and antibodies (anti-HBc Total II, HBc IgM, Anti-HBsT II) was performed by Enzyme Linked Fluorescent Assay (ELFA) in serum samples of 878 pregnant women attended at the Lucrecia Paim Maternity Hospital (LPMH). Data were collected by questionnaire after written consent, and spatial distribution was assessed through a Kernel Density Function. The potential risk factors associated with HIV HBV infection were evaluated using bivariate and multivariate binomial logistic regression analysis.

**Results:**

Anti-HIV antibodies were positive in 118 samples (13.4%) and HBV infection were positive in 226 (25.7%). The seroprevalence of HIV/HBV coinfection was of 6.3%. The results showed that the seroprevalence of HBV was similar in most municipalities: 25.8% in Belas; 26.6% in Viana; 27.6% in Luanda; 19.2% in Cacuaco; and 15.6% Cazenga. For HIV, the seroprevalence was also close ranges among the municipalities: 10.0% in Belas; 14.5% in Viana 14.9% in Luanda and 12.5% in Cazenga. However, the seroprevalence in municipality of in Cacuaco was lower (5.8%) and bivariate and multivariate analysis showed a lower risk for HIV in this area (OR 0.348, CI 0.083–0.986; OR 0.359, CI 0.085–1.021). The multivariate analysis had also showed a significant increased risk for HIV in women with 2 or 3 births (OR 1.860, CI 1.054–3.372).

**Conclusions:**

Our results underlined the need to improve the screening and clinical follow-up of HIV and HBV in Angola, as well the educational campaigns to prevent not only the morbidity and mortality associated with these diseases, but also their transmission, mainly in women in reproductive age and pregnant, encouraging the pre-natal consultations in order to avoid mother-to-child transmission.

**Supplementary Information:**

The online version contains supplementary material available at 10.1186/s12985-021-01698-7.

## Introduction

Infectious diseases alter women's health and may negatively influence their reproductive function, which is why they continue to be a public health concern, especially in developing countries [1, 2]. When associated with pregnancy, infectious diseases take on special importance and pose three particular issues: the treatment of the mother's disease; the effect of infection in the course of pregnancy; and the influence on the fetus not only of maternal disease, but also of the therapy used.

The Human Immunodeficiency Virus (HIV) lead to the progressive deterioration of the immune system, which promotes the development of opportunistic infections. Additionally, the HIV and Hepatitis B Virus (HBV) are the main cause of chronic viral infections globally and are among the infections with importance and prominence in the clinical evaluation of a pregnancy [3, 4]. HBV infection in pregnancy has serious implications, including increasing the risk of development of chronic HBV, perinatal transmission of HBV and accelerated HBV-related liver damage [5].

The World Health Organization (WHO) Global Health Observatory (GHO), reports that approximately 36.9 million people are infected with HIV worldwide, more than half of whom are women and 1.8 million children [6]. In Africa, HIV infection rates in pregnant women range from 15 to 40% with the highest global HIV prevalence in women of reproductive age, representing more than 55% of adults infected with the virus [7]. Moreover, according to the Global Hepatitis Report [3] it was estimated that 257 million people (3.5%) lived with chronic HBV infection worldwide. About 68% of infected people live in African and Western Pacific regions [8]. The prevalence rates of HBV infection and HIV–HBV coinfection in pregnancy in Africa range from 6.3 to 25% [9-11] and from 0.7 to 11.6% respectively [12-16]. Almost 2.7 million people have been co-infected with HBV and HIV and Sub-Saharan Africa is the epicenter of many infectious diseases, including HIV and HBV [5, 17, 18].

Co-infection with HIV/HBV can happen vertically from mother to child or horizontally through exposure to the infected blood and also other body fluids [19, 20]. The risk of vertical transmission of HBV is greater among women living with HIV compared to those who are HIV-negative [21]. Furthermore, HBV-infected infants born to HBV/HIV co-infected mothers are at higher risk of hepatic complications and death compared to those born to HBV mono-infected women [21]. Vertical transmission can be averted with antiviral treatment of pregnant women (for prevention of both HIV and HBV) and infant vaccination (for prevention of HBV) [22].

Screening for these infections during pregnancy can reduce the risk of vertical transmission to less than 5%; however, without intervention, vertical transmission can be around 15–45% [3].

In order to implement appropriate mother-to-child prevention strategies among women living with HIV, it is important to first determine the prevalence of HIV/HBV co-infection. Despite the endemicity of HBV in Angola and the knowledge that its clinical course is worsened by co-infection with HIV, there is very little epidemiological data on HBV/HIV confection. Also, no research to date has explored the overall seroprevalence for HBV infection among women in Angola, nor have the risk factors associated with the infection been examined in a regional context.

Therefore, the objective of this study was to determine the seroprevalence of HIV and HBV in pregnant women who attended a referral maternity facility located in Luanda (Angola), and to provide a detailed analysis of the geographical distribution. The study also evaluated the influence of demographic variables, socio-economic features, and hygiene and health conditions on the HIV and HBV infection. This knowledge is essential for the development of effective prevention and control strategies and the implementation of the HIV and HBV Surveillance Program in the Angolan National Health System network.

## Material and methods

The present study has been approved by the Research Ethics Committee of Lucrécia Paim Maternity Hospital (LPMH) through the National Institute of Public Health of the Republic of Angola (nº 301,019; Additional file 1: S1 File). Participating individuals provided a written signed informed consent prior to sample collection and for participants younger than 18 years, informed consent was provided by parents or guardians after a detailed explanation of the objectives of the work.

### Ethical considerations

The Republic of Angola is located on the west coast of sub-Saharan Africa. It is one of the largest countries of the continent, with a surface of 1,246,700km2 and near 25.8 million inhabitants [23]. Angola, like most developing countries, has a fairly young population. The population aged 0–14 years is 12,196,496, representing 47% of the total resident population [23]. It is estimated that there are 48% of men and 52% of women and that children fewer than 5 years old constitute 15% of the total population [23]. The economically active population is less than 50%, with a large number of state and family dependents. Women of reproductive age (15–49 years) make up about 44% and the estimated fertility rate is 6.2 children per woman [24].

### Study area

Luanda is the capital and largest city in Angola, located on the coast with the Atlantic Ocean, is also the primary port and economic center of the country. According to the last census conducted in 2014, Luanda has a population of 6,945,386 inhabitants and is composed of 7 municipalities: Belas, Cacuaco, Cazenga, Ícolo and Bengo, Luanda, Quissama and Viana [23].

## Study population

The study population was constituted by pregnant women monitored for routine prenatal assessment at LPMH, a reference maternity in Luanda, the capital of Angola. The maternity is a tertiary-level public health institution specialized in maternal and child health care, teaching and research. The health institution offers outpatient and inpatient services, has 400 beds for hospitalization video laparoscopy, hysteroscopy, milk bank, pathological anatomy, assisted reproductive services, genetics and mammography. Consequently, the women attended in the LPMH come from all over the country, however mainly from Luanda. The study included women aged from 15 to 47 years, who had a pregnancy proven by ultrasonography and laboratory tests. For the obstetric follow-up we counted on the collaboration of the medical and nursing team of the department of obstetrics of the LPMH.

Standardized questionnaire in face-to-face interviews were used to obtain the socio-demographic, clinical, and housing characteristics from the pregnant women (Additional file 2: S2 File). Socio-demographic items included age, residence, occupation, schooling level, and socio-economic status. Clinical characteristics included gestation age, number of pregnancies, history of abortion, the frequency of miscarriages and presence of any underlying disease. The questionnaire was written in Portuguese, the official language in the Republic of Angola, developed and revised accordingly.

### Blood sample collection and laboratory procedures

A cross-sectional survey was carried out from August 2016 to May 2017. The study included pregnant women from all trimesters (n = 878). From each pregnant woman venous blood was collected and serum samples were obtained after centrifugation. These serum samples were immediately transferred (properly packaged in dry ice) to the Clinical Pathology Service of Clínica Sagrada Esperança (Luanda) and kept at -80 °C until serological analysis. Detection of anti-HIV antibodies (total anti-HIV-1, anti-HIV-2) and HIV-1 p24 antigen was performed by Enzyme Linked Fluorescent Assay (ELFA) using commercial kits for VIDAS (HIV DUO Ultra, HIV5 and HIV P24 II) (Biomerieux, Portugal). The Hepatitis B infection was characterized by the detection of surface antigen HBs Ag. The HBsAg positive samples were retested for HBeAg and antibodies Anti-HBc Total I and HBc IgM, using a commercial kits ELFA for VIDAS (Biomerieux, Portugal). The profile HBsAg positive/HBc IgM negative define a chronic HBV infection.

### Geospatial analysis

The address of pregnant women was collected during the interview allowing the identification of the residence place. This information was converted into geographic coordinates (latitude and longitude) through the www.google.pt/maps/. The spatial distribution of pregnant women was assessed through a Kernel Density Function that allowed the estimation of the intensity of events across a surface (Additional file 3: S3 File).

## Statistical analysis

The data entry was carried out using Excel software and analysed through Statistical Package for the Social Sciences (SPSS) version 20. The exploratory analysis of the categorical variables and quantitative variables are presented as percentages (mean ± SD). Bivariate and multivariate logistic regressions were developed to assess the effect of different risk factors on HIV seroprevalence. The level of statistical significance was set as *p* < 0.05, and Odds Ratio (OR) and 95% Confidence Intervals (CI) were computed.

## Results

### Seroprevalence and Sociodemographic characteristics

Between August 2016 and May 2017, a total of 878 pregnant women were tested for anti-HIV antibodies and HBsAg. The distribution of antibodies to HIV and HBsAg of seropositive and seronegative pregnant women is summarized in Table 1. The majority of women had not exposure to the HIV: 760 (86.6%) were negative for anti-HIV; and 118 (13.4%) were positive for anti-HIV antibodies. For HBsAg, 226 (25.7%) were positive and 652 (74.3%) were negative (Table 1).

**Table 1:** Distribution of antibodies for HIV and HBS antigen (HBsAg) of seropositive and seronegative pregnant women from Luanda (Angola)

Seroprevalence	Positive (%)	Negative (%)
Anti-HIV	118 (13.4)	760 (86.6)
HBsAg	226 (25.7)	652 (74.3)

Of the 226 HBsAg positive participants,148 (65.5%) were positive for Total HBc and negative for HBc IgM, and 78 (34.5%) were positive for HBeAg. The seroprevalence of HIV/HBV coinfection was of 6.3%. The majority of women are susceptible to the infections.

The characteristics of the individual pregnant women and details on home conditions were collected using a structured questionnaire (Additional file 1: S1 File) and summarized in Table 2. The age of surveyed women ranged from 15 to 47 years, with a mean of 29.03 ± 5.6 years; pregnant women between 26 and 35 years were the majority of participants (n = 504) (Table 2). Regarding education level, 9 (1.0%) are illiterate, 341 (38.9%) have basic education, 439 (50.0%) have a high school education and 89 (10.1%) have higher education.

**Table 2:** Sociodemographic characteristics of seropositive and seronegative pregnant women to HIV and HBsAg

Characteristics	Anti-HIV		HBsAg		Total
	Positive n (%)	Negative n (%)	Positive n (%)	Negative n (%)	
*Age group (years)*					
≤ 19	3 (2.5)	37 (4.9)	5 (2.2)	35 (5.4)	40 (4.6)
20–25	29 (24.6)	164 (21.6)	47 (20.8)	146 (22.4)	193 (21.9)
26–35	61 (51.7)	443 (58.3)	141 (62.4)	363 (55.7)	504 (57.4)
36–47	25 (21.2)	116 (15.2)	33 (14.6)	108 (16.5)	141 (16.1)
*Education*					
Illiterate	1 (0.8)	8 (1.0)	3 (1.3)	6 (0.9)	9 (1.0)
Elementary School	51 (43.2)	290 (38.2)	82 (36.3)	259 (39.8)	341 (38.9)
High school	58 (49.2)	381 (50.1)	118 (52.2)	321 (49.2)	439 (50.0)
Higher education	8 (6.8)	81 (10.7)	23 (10.2)	66 (10.1)	89 (10.1)
*Marital status*					
Married	41 (34.7)	241(31.7)	71 (31.4)	211(32.4)	282 (32.1)
Single	77 (65.3)	519 (68.3)	155 (68.6)	441 (67.6)	596 (67.9)
*Occupation*					
Homemakers	25 (21.2)	176 (23.2)	64 (28.3)	137 (21.0)	201 (22.9)
Public function	39 (33.1)	242 (31.8)	67 (29.6)	214 (32.8)	281 (32.0)
Student	24 (20.3)	167 (22.0)	47 (20.8)	144 (22.1)	191(21.8)
Restaurant waitress	19 (16.1)	92 (12.1)	26 (11.5)	85 (13.0)	111(12.6)
Street vendor	6 (5.1)	48 (6.3)	8 (3.6)	46 (7.1)	54 (6.1)
Store clerk	5(4.2)	35 (4.6)	14 (6.2)	26 (4.0)	40 (4.6)
*Residence*					
Belas	12 (10.2)	108 (14.2)	31 (13.7)	89 (13.7)	120 (13.7)
Cacuaco	3 (2.5)	49 (6.4)	10 (4.4)	42 (6.4)	52 (5.9)
Viana	30 (25.4)	177 (23.3)	55 (24.4)	152 (23.3)	207 (23.6)
Cazenga	8 (6.8)	56 (7.4)	10 (4.4)	54 (8.3)	64 (7.3)
Luanda	65 (55.1)	370 (48.7)	120 (53.1)	315 (48.3)	435 (49.5)
*Awareness of HIV and Hepatitis B*					
Does not know anything	3 (2.5)	8 (1.0)	3(1.3)	8 (1.2)	11 (1.2)
Heard about it but doesn't know anything	107 (90.7)	705 (92.8)	214 (94.7)	598 (91.7)	812 (92.5)
Know anything about the disease	8 (6.8)	47 (6.2)	9(4.0)	46 (7.1)	55 (6.3)
*Access to basic sanitation*					
Yes	78(66.1)	480 (63.2)	148(65.5)	410 (62.9)	558 (63.6)
No	40 (33.9)	280 (36.8)	78 (34.5)	242 (37.1)	320 (36.4)
*Gestational age*					
1st Trimester	37(31.4)	238 (31.3)	72(31.9)	203 (31.1)	275 (31.3)
2nd Trimester	44 (37.2)	334 (44.0)	97 (42.9)	281 (43.1)	378 (43.1)
3rd Trimester	37 (31.4)	188 (24.7)	57 (25.2)	168 (25.8)	225 (25.6)
*History of abortion*					
No	62 (52.5)	416 (54.7)	104 (46.0)	296 (45.4)	478 (54.4)
Yes	56 (47.5)	344 (45.3)	122 (54.0)	356 (54.6)	400 (45.6)
*Number of births*					
0	24 (20.4)	198 (26.0)	57 (25.2)	165(25.3)	222 (25.3)
1	28 (23.7)	214 (28.2)	67(29.7)	175 (26.8)	242 (27.6)
≥ 2	66 (55.9)	348 (45.8)	102 (45.1)	312 (47.9)	414 (47.1)
*Spontaneous abortion*					
Yes	4 (3.4)	41 (5.4)	9 (4.0)	36 (5.5)	45 (5.1)
No	114 (96.6)	719 (94.6)	217 (96.0)	616 (94.5)	833 (94.9)
*Pre-natal consultation was performed in all pregnancies*					
Yes	3 (2.5)	22 (2.9)	221 (97.8)	633 (97.1)	854 (97.3)
No	115 (97.5)	738 (97.1)	5 (2.2)	19 (2.9)	24 (2.7)
*Recent blood transfusion*					
Yes	3 (2.5)	4 (0.5)	3 (1.3)	4 (0.6)	7 (0.8)
No	115 (97.5)	756 (99.5)	223 (98.7)	648 (99.4)	871 (99.2)
*Recently needle-stick injury or sharp objects*					
Yes	3 (2.5)	22 (2.9)	5 (2.2)	20 (3.1)	25 (2.8)
No	115 (97.5)	738 (97.1)	221 (97.8)	632 (96.9)	853 (97.2)
Total	118	760	226	652	878

Concerning occupation, 201 (22.9%) are homemakers, 281 (32.0%) women work on public administration services, 191(21.8%) are students, 111(12.6%) work as restaurant waitress, 54 (6.1%) are street vendors, and 40 (4.6%) are store employees. The majority of pregnant women 596 (67.9%), are single and 282 (32.1%) are married. Regarding gestational age, 275 (31.3%) were in the 1st trimester, 378 (43.1%) in the 2nd trimester and 225 (25.6%) in the 3rd trimester respectively. The mean gestational age was 19.8 ± 9.4 weeks (Mean ± SD; median = 16). Concerning the number of births, 222 (25.3%) had no children, 242 (27.6%) had one children, 414 (47.1%) had two or more children. No children were born prematurely although 45 (5.1%) women suffered spontaneous abortion while the present study was under way (Table 2).

In relation to blood transfusions, only 7 (0.8%) pregnant women reported having recently had a blood transfusion and 25 (2.8%) reported having recently been subjected to a needle-stick injury (Table 2). More than half of the participants 558 (63.6%) reported having basic sanitation at home.

Most of the pregnant women, 812 (92.5%) reported lack of knowledge of the virus diseases under study, 55 (6.3%) had hear of HIV and HBV but they do not know anything about it and 11 (1.2%) pregnant women reported knowing something about the diseases under study. Additionally, 854 (97.3%) women stated that they were monitored for routine prenatal assessment in all their pregnancies and 478 (54.4%) reported history of miscarriage and there was no child death in the postpartum period (Table 2).

The frequency of positivity for anti-HIV antibodies was higher among pregnant women in the second trimester 37.2% (n = 44), followed by pregnant women in the first 31.4% (n = 37) and third trimester 31.4% (n = 37). The frequency of HIV infection in relation to the pregnant women age was 2.5% (n = 3) in the group of ≤ 19 years, 24.6% (n = 29) in the group of 20–25 years, 51.7% (n = 61) in the group of 26–35, and 21.2% (n = 25) in the group of 36–47 years (Table 2). Regarding the obstetric history, the frequency of HIV infection was similar in pregnant women regardless of the month of gestation and parity (number of births or miscarriages).

The frequency of HBsAg positivity was also higher among pregnant women in the second trimester 42.9% (n = 97) followed by pregnant women in the first trimester 31.9% (n = 72) and in the third trimester 25.2% (n = 57) (Table 2). The frequency of positivity the HBsAg in relation to the age of pregnant women was 2.2% (n = 5) in the group ≤ 19 years, 20.8% (n = 47) in the group of 20–25 years, 62.4% (n = 141) in the group of 26–35 years and 14.6% (n = 33) in the group of 36–47 years (Table 2).

Relatively to the prevalence by municipalities, it was found that Luanda municipality had a greater number of seropositive pregnant women for HBsAg (120 of 226 positive cases; 53.1%) and for HIV (65 of 118 positive cases; 55.1%), probably because it has the larger population surveyed (435 of 878; 49.5%) (Table 2). The other municipalities had 106 positive cases (46.9%) for HBsAg and 53 positive cases (44.9%) for HIV virus.

### Associated risk factors of HIV and HBV infection

The multivariate logistic regression analysis (adjusted by age) revealed a statistically significant association between HIV infection and the number of births, where woman with 2 or 3 births present 86% higher risk of having HIV than woman with no previous births (*p* = 0.0357) (Table 3). Women having age ≥ 30 years had higher odds of HIV positivity (OR = 1.20, 95% CI = 0.675–2.123) as compared with those ≤ 29 years. Area of residence is not a significant risk factor for HIV infection.

**Table 3:** Binomial logistic regression models for the final analysis of risk factors associate for seropositivity of anti-HIV antibodies in 878 pregnant women in Luanda province, Angola

Variables	OR (95%CI)	*p* value	OR (95%CI)	*p* value
	Unadjusted		Adjusted by age	
Age range (Ref = ≤ 29 years old)				
≥ 30 years old	1. 2048 (0.6750- 2.1234)	0.522		
Education (Ref = High (high school or higher education)				
Low (up to elementary school)	1.2214 (0.8233–1.8044)	0.317	1.1825 (0.7948–1.7512)	0.404
Occupation (Ref = Homemakers**)**				
Student	1.0117 (0.5537–1.8451)	0.970	0.9545 (0.5183–1.7537)	0.881
Public function	1.1345 (0.6660–1.9636)	0.646	1.1068 (0.6484–1.9186)	0.713
Street vendor, store and catering	1.2068 (0.6829–2.1486)	0.518	1.1436 (0.6441–2.0444)	0.647
Marital status (Ref = Single)				
Married	1.1466 (0.7566–1.7165)	0.511	1.1711 (0.7717–1.7561)	0.450
Gestational age				
Gestational age (Ref = 1st trimester)				
2nd and 3rd trimester	0.9981 (0.6615–1.5299)	0.993	0.9824 (0.6499–1.5085)	0.934
Number of births (Ref = 0)				
1	1.0794 (0.6056–1.9370)	0.7956	1.2035 (0.6655–2.1934)	0.5406
2 or 3	1.5646 (0.9625–2.6202)	0.0785	1.8608 (1.0549–3.3728)	0.0357*
Spontaneous abortion (Ref = No)				
Yes	0.6153 (0.1823–1.5602)	0.363	0.6338 (0.1868–1.6212)	0.396
History of abortion (Ref = No)				
Yes	0.9155 (0.6208–1.3525)	0.656	0.8785 (0.5401–1.4418)	0.604
Access to basic sanitation (Ref = Yes)				
No	0.8791 (0.5797–1.3154)	0.537	0.8643 (0.5691–1.2948)	0.486
Recently needle-stick injury or sharp objects (Ref = No)				
Yes	0.8750 (0.2048–2.5770)	0.831	0.8955 (0.2092–2.6458)	0.860
Residence (Ref = Luanda)				
Belas	0.6324 (0.3152–1.174)	0.1685	0.6259 (0.3115–1.1638)	0.1600
Cacuaco	0.3485 (0.0830–0.9869)	0.0839	0.3598 (0.0856–1.0219)	0.0943
Viana	0.9647 (0.5973–1.5284)	0.8808	0.9834 (0.6081–1.5606)	0.9444

OR: Odds ratio, CI: confidence interval

*Statistically significant (*p* < 0.05)

Live in Cacuaco (OR = 0.348; 95% CI = 0.083–0.986), had a spontaneous abortion (OR = 0.615; 95% CI = 0.182–1.560), needle-stick injury or sharp objects (OR = 0.875; 95% CI 0.204–2.577) as well as longer gestational age (OR = 0.998; 95% CI 0.661–1.529) were found to be associated with lower odds HIV infection, although not significantly (p > 0.0.05).

The odds of HIV infection were 1.146 higher among women married compared to singles (95% CI 0.756–1.716) (Table 3). In relation with occupation, women who reported to be street vendor, store clerk and work on restoration (OR = 1.206; 0.68– 2.148), public function (OR = 1.134; 95% CI 0.666–1.963), and student (OR = 1.117; 95% CI 0.553–1.845) had higher prevalence as compared with homemakers.

**Fig. 1:**
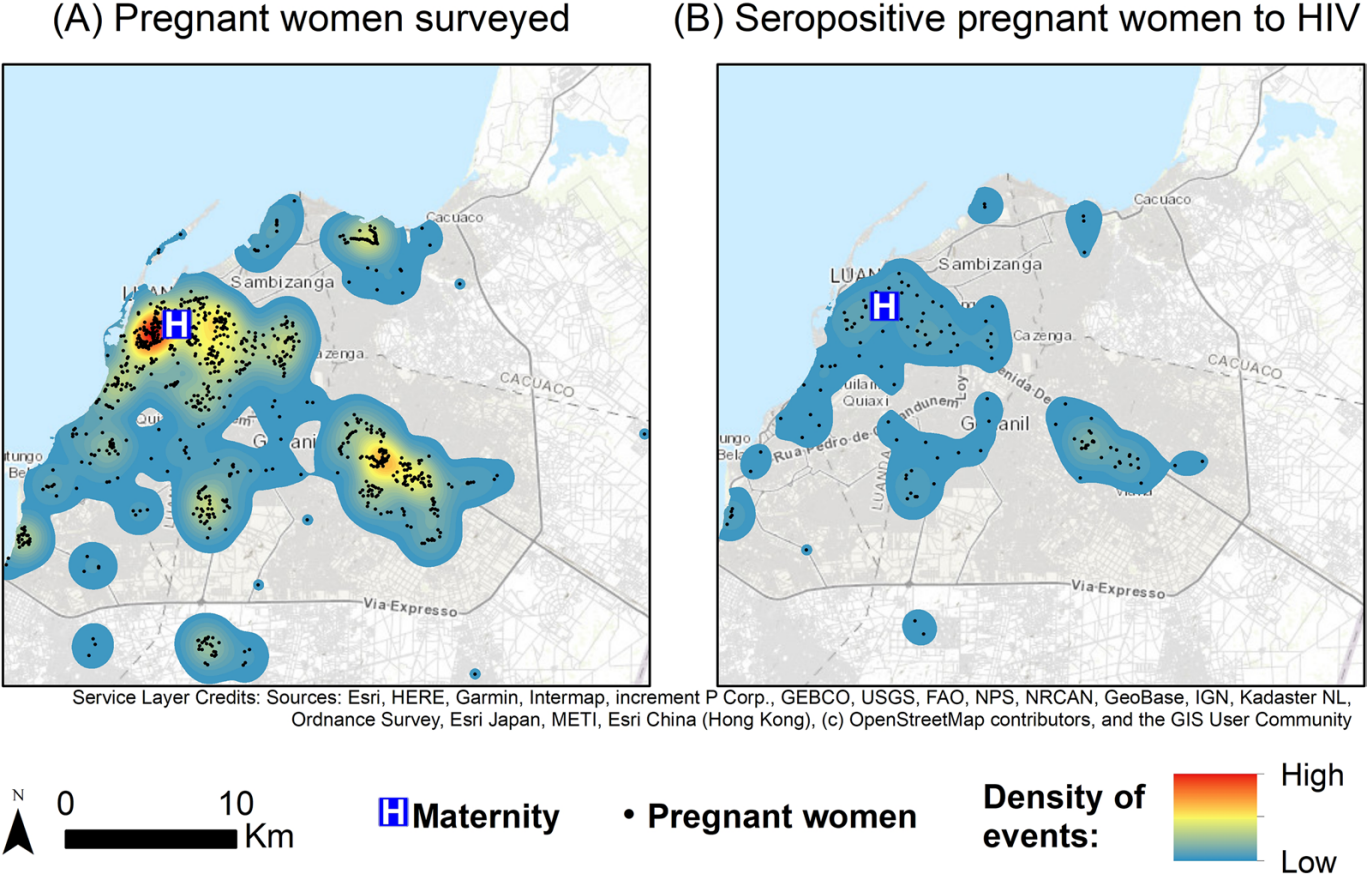
Geographical distribution and Gaussian kernel density surface map of survey pregnant women (**A**) and with positive anti- HIV antibodies (**B**) in Luanda, Angola

**Fig. 2:**
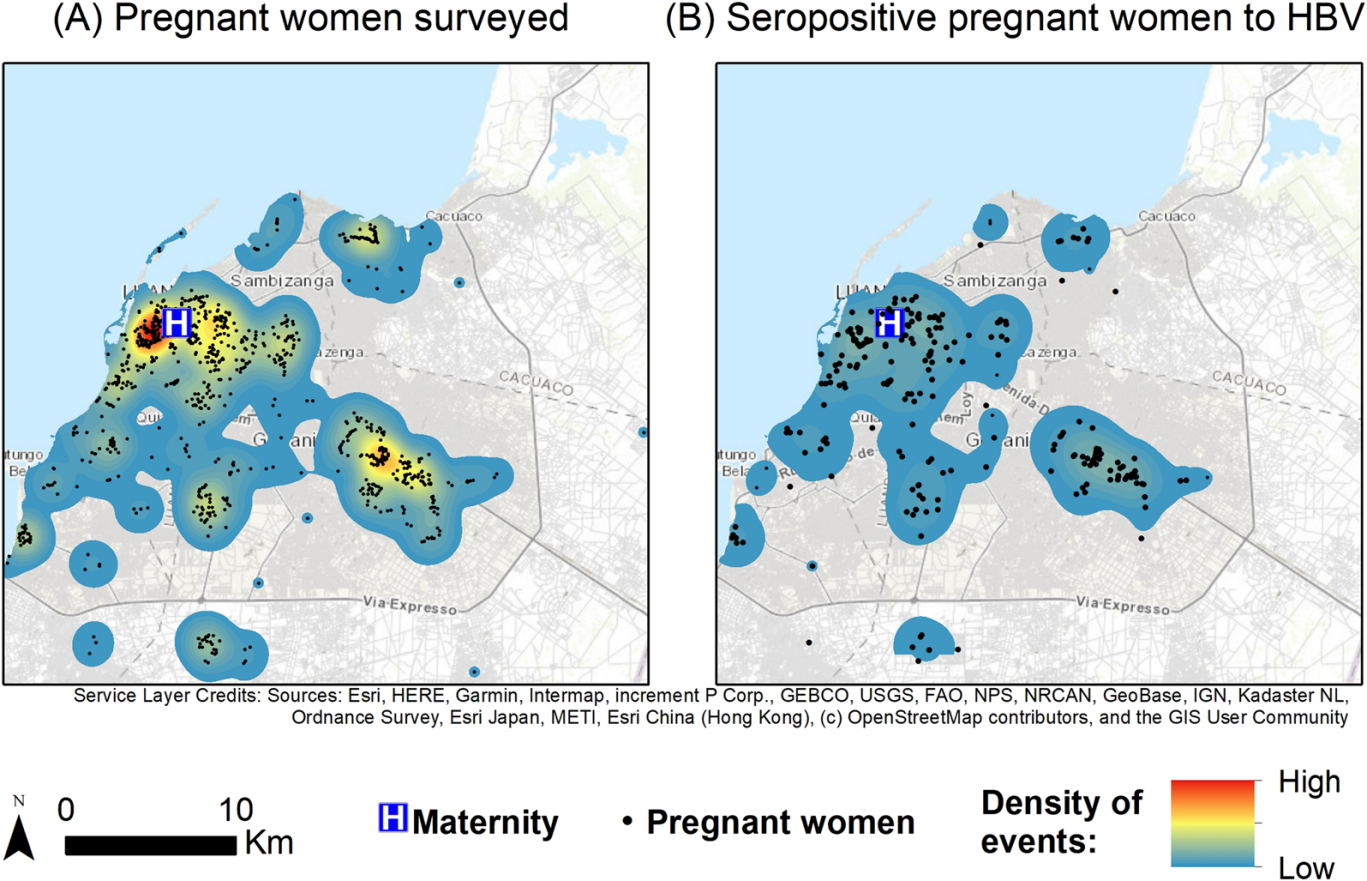
Geographical distribution and Gaussian kernel density surface map of survey pregnant women (**A**) and with positive HBsAg (**B**) in Luanda, Angola

The spatial pattern of pregnant women and seropositive pregnant women to HIV and to HBV is presented in Figs. 1 and 2, respectively, through a density map. It can be observed that the patterns presented are similar, with more events in Luanda City center, around the hospital LPMH, followed by a hotspot in Viana. The surroundings of Luanda do not present records of pregnant women admitted at the hospital.

## Discussion

The present study was performed to investigate the seroprevalence of HIV and HBV infection in pregnant women in the province of Luanda evaluating some epidemiological aspects and identifying the risk factors in the population. Spatial analysis was applied for the first time to the case of Luanda to describe the geographical distribution of HIV and HBV in pregnant women. To measure seroprevalence of HIV and the HBV, anti- HIV antibodies quantification were performed and in the case of HBV the quantification of HBsAg and HBeAg on pregnant women attending the LPMH from August 2016 to May 2017.

The seroprevalence of antibodies anti-HIV in our study was 13.4%, which is similar with the studies carried out in Ethiopia (10.1%) [25], Zambia (11.3%) [26] and Democratic Republic of Congo (11%) [27]. However, it was higher than previous studies carried out in Nigeria (6.1%) [28], Cameroon (6%) [29], Tanzania (5.1–5.6%) [30, 31], Brazil (0.37%) [32], Dominican Republic (5.8%) [33], and India (0.88%) [34].

Regarding prevalence in Luanda, our results showed that HIV infection among pregnant women is higher when compared to a sentinel study carried out in 2003 (4.5%) [35] and a survey in 2018 (2.6%) [36]. The difference could be related with the method that we used in quantification of anti- HIV antibodies (Enzyme-linked fluorescence assay) with higher sensitivity and specificity than rapid diagnostic tests. Moreover, attending that in our study only 6.3% of pregnant reports awareness of HIV, the prevalence observed in our study could also be related with the lack of knowledge of disease and how to prevent them. On the other hand, 2018 cohort was almost twice as large (n = 1612) than ours (n = 878) and so could partially explain the difference on obtained results.

The differences in HIV prevalence rates in the worldwide population may be associated with various factors related to each region/country and specific characteristics of the population as well with the diagnostic methods used. Other examples include sanitary practices, socioeconomic status and lack of knowledge about the disease.

Globally, the hepatitis B prevalence in adults is highest in the WHO Western Pacific Region (6.2%) and the WHO African region (6.1%). In Sub Saharan Africa, the overall HBsAg carrier rate in the general population is 5–20%, which is among the highest in the world [37]. Perinatal transmission is estimated at 1–5% [38]. But, despite the high endemicity of HBV infection in sub-Saharan Africa, little is known about HBV seroepidemiology and genotype distribution in many African countries, such as Angola [39].

The first published study about HBV epidemiology in Angola have more than 25 years ago and was carried out in general population, showing a high prevalence of HBV (79%) [40]. Other study, published eleven years ago, confirmed the extremely high HBV rates: 79.7% of the studied population was anti-HBc positive, which indicates previous contact with the virus; and 15.1% of the individuals were carriers of HBsAg, which is a marker of active HBV infection [39]. In Angola, the National Vaccination Program includes the HBV vaccine at birth [41]. Today, little is known about prevalence at the level of the Luanda general population, and consequently the real number of Angolans exposed to HBV. On the other hand, also it is not known how many pregnant women is hepatitis B positive at national level.

The seroprevalence of HBV in our study was 25.7%, relatively high value that has been consistently reported by several studies conducted in similar populations [42-45]. In contrast, this prevalence of HBsAg was higher than prevalence rates reported in Nigeria (8.3%) [46], Ethiopia (6.9%) [47] and in Mali (8.0%) [14]. Also the prevalence achieved in the present study is higher when compared to a study performed recently in Luanda (7.5%) [36]. This difference might be related with the method used by us in the quantification of HBsAg (ELFA) which has a high sensitivity and specificity. Moreover, we evaluated HBV infection in all pregnant women who attend external consultations without any exclusion. The application of exclusion criteria may have skewed the participating population compared with general obstetric population.

Overall the differences observed in the countries might be attributable to differences in the study population [45], cultural practices [44], and the obvious natural difference linked with various geographical situations [25].

The HBeAg status and the HBV viral load are both factors known to be associated with vertical HBV transmission [22, 48]. We have assessed the presence of HBeAg which is a marker of high infectivity, and an excellent marker for measuring the risk of vertical transmission of HBV. Out of 226 HBsAg positive pregnant women, 78 (34.5%) were positive for HBeAg. This finding was similar to that of a study carried out in Ethiopia [25] but, significantly higher as compared to other studies elsewhere [49, 50]. This might be due to difference in diagnostic methods which were used. It is known that the risk of vertical transmission and resulting chronic infection from HBsAg (+) mother to her baby is approximately 90% in HBeAg-positive pregnant women [51]. A recent study concluded that vertical transmission is an important means of HBV transmission in areas where there is no birth dose vaccination program for newborn of HBsAg carrier mothers [25]. It is known that vertical transmission of HBV, either in utero or peripartum, is responsible for up to 50% of HBV infection worldwide [29] and that the risk of transmission can be reduced by 90% when vaccine coupled with HBV Immune Globulin are administered to the infant at birth [52, 53].

Our study found an HIV/HBV co‐infection prevalence of 6.2% among pregnant women living in Luanda Province. This prevalence value is not surprising since the HIV/HBV co‐infection in sub‐Saharan Africa is estimated at 6% to 25% [54]. Our finding is similar with those reported in HIV‐positive pregnant women from Cameroon (7.7%) [55], Botswana (3.1%) [56], Rwanda (4.1%) [57], but lower than those seen in Ethiopia (12.1%) [58], and Ghana (14.9%) [4], and Nigeria (12.6%) [5]. Most of the time, high HIV/HBV co‐ infection is attributed to the fact they share mutual routes of transmission. The high HIV/HBV co‐infection in pregnant women suggests a potential source for the spread of viral infections in Luanda. Moreover, it should be particular attention to HIV/HBV co-infection in pregnancy because may alter the selection of antiretroviral drug regimens to those with dual action against both viruses.

There is evidence supported that infectious diseases are associated to the socioeconomic and health indicators of a given country. The inequalities of resource distribution in the population unemployed and with low educational levels lead to risky sexual behavior and an increase in infectious diseases [1, 36]. There exist few national studies reported about the impacts of regional and age differences on clinical epidemiology of HIV/AIDS and the HBV in Angola [24, 59, 60].

In our multivariate analysis, there is an association between HIV infection and women with 2 or 3 previous births. Also the women´s married and with employment had higher odds of HIV positivity. In contrast, lives in Cacuaco is associate with low odds of HIV positivity. In relation to HBV infection, sociodemographic variables like age, marital and educational status, residence, and occupation of participants as well as reproductive variables like gestational age were not significantly associated with the risk of infection. This finding is in line with the study conducted in Ethiopia [61] and Nigeria [62]. In contrast, other studies showed that pregnant women with no formal education had higher odds of HBV infection [63].

HIV and HBV infections remain responsible for high rate of morbidity and mortality in many African Countries, affecting women and infants born from infected mothers [22].

## Conclusions

Routine screening for HIV and HBV infection during pregnancy is performed for most Angolan women, as the test is mandatory in prenatal care. However, poor access to higher education and the lack of promotion of public health in general explain how a very high percentage of the population lacks basic knowledge of HIV and HBV infections. Therefore, it is crucial to include education on HIV and HBV prevention in prenatal counseling, as well other infectious diseases, to enable the diagnosis of maternal infection and avoid vertical transmission. More studies should be performed to identify the risk factors for HIV and HBV in Angola, including surveys carried out in the general population.

## Supplementary Information

**Additional file 1**. **File S1**. Ethics Committee, República de Angola, Ministério da Saúde.

**Additional file 2**. **File S2**. Recruitment Questionnaire/Questionário de Recrutamento.

**Additional file 3**. **File S3**. Programs and datasets used to create the maps.

## Data Availability

Authors declare that all data are fully available without restriction.

## Abbreviations

AIDS: Acquired immunodeficiency syndrome
CEGOT: Centre of studies on geography and spatial planning
CI: Confidence intervals
CNC: Center for neuroscience and cell biology
ELFA: Enzyme linked fluorescent assay
FFUC: Faculty of Pharmacy, University of Coimbra
GHO: Global Health Observatory
HBeAg: Hepatitis B e antigen
HBsAg: Hepatitis B surface antigen
HBV: Hepatitis B Virus
HIV: Human Immunodeficiency Virus
LPMH: Lucrécia Paim Maternity Hospital
OR: Odds ratio
PMTCT: Prevention of mother-to-child transmission
WHO: World Health Organization
