# Supplementary material for: Prevalence of HIV and hepatitis B virus among pregnant women in Luanda (Angola): geospatial distribution and its association with socio-demographic and clinical-obstetric determinants
Source: Virol J. 2021 Dec 4;18:239. doi: 10.1186/s12985-021-01698-7 (PMC8642931; doi:10.1186/s12985-021-01698-7)
Supplement: Supplementary file 2 — Additional file 2. File S2. Recruitment Questionnaire/Questionário de Recrutamento. [file 12985_2021_1698_MOESM2_ESM.pdf]

QUESTIONNAIRE

Date ___/___/___

Internal Number:_________

Laboratory: __________________________

Process number: _________________

Personal data

Residence: _________________

Level of education: _________

Age: ______________ Gestational Age: ___________

Number of births

0 ☐

1 and 2 ☐

≥3 ☐

Did you do prenatal consultation in all pregnancies?

No ☐

Yes ☐

<u>Risk factors</u>

1. Have you ever done a HIV and Hepatitis B virus (HBV) test?

No ☐

Yes ☐

2. Awareness of HIV and Hepatitis B:

Do not you know anything about the diseases ☐

It has hear spoken, but does not know anything about it ☐

know anything about the diseases ☐

3. In the residence, do you have a basic sanitation? (water treated, collection refuse, sewage system):

Yes ☐

No ☐ If not, which one do not have?

Water treated ☐

Collection refuse ☐

Sewage system ☐

4. Do you currently have pets at home?

No ☐

Yes ☐ If so, which? Cats ☐

Dogs ☐

Others ☐

5. Currently has contact with animals other than yours at home the of relatives or other households.?

No ☐

Yes ☐ If so, which?

Cats ☐

Dogs ☐

Others ☐

6. Do you have contact with cats other than yours?

No ☐

Yes ☐ If so, how often?

Very frequent ☐

Less frequent ☐

Rarely ☐

7. In the case you have pet cats, where do they defecate?

Inside home ☐

In the vicinity of the house ☐

Far from the house ☐

8. If you have pet cats, what is your main food?

Ration ☐

Leftover food (cooked) ☐

Restos de carne crua ☐

9. Do you know of rodents at or near your home?

No ☐

Yes ☐

10. Do you have a garden in your residence?

No ☐

Yes ☐ In the case of yes this is fenced, preventing the entry of cats

No ☐

Yes ☐

11. Do you carry out soil-related activities (gardening, agriculture, etc.)?

No ☐

Yes ☐

12. Do you have farmed animals for your own consumption?

No ☐

Yes ☐ if yes, which one

Cattle ☐

Pigs ☐

Birds ☐

Others ☐

12. Do you have habits of consuming meat from animals slaughtered in hunting such as birds, rabbits, wild boars etc.

No ☐

Yes ☐

13. Do you eat raw or undercooked meat?

No ☐

Yes ☐ if yes, from which animals?

Cattle ☐

Pigs ☐

Birds ☐

☐

Others

if yes, how often?

Very common ☐

Less common ☐

Rarely ☐

14. Do you consume unpasteurized milk or dairy products?

No ☐

Yes ☐

if yes, how often?

Very common ☐

Less common ☐

Rarely ☐

15. Do you always wash fruits and vegetables before consuming them?

No ☐

Yes ☐

if yes, how often?

Very common ☐

Less common ☐

Rarely ☐

16. Do you eat raw or undercooked eggs?

No ☐

Yes ☐

if yes, how often?

Very common ☐

Less common ☐

Rarely ☐

17. Do you consume water from well / borehole?

No ☐

Yes ☐ if yes, how often?

Very common ☐

Less common ☐

Rarely ☐

18. Have you recently had a blood transfusion?

No ☐

Yes ☐

19. Recently had you a needle stick / syringe accident?

No ☐

Yes ☐

Questionário de Recrutamento

Data ___/___/___

Número interno:_________

Laboratório: ___________________________

Número do Processo: _________________

Dados Pessoais

Residência: _________________

Nível de escolaridade: _________

Idade: ______________Idade Gestacional: ___________

Quantas vezes já esteve grávida?

Uma ☐

Duas ☐

Mais ☐

Fez consulta pre - natal em todas as gestações?

Não ☐

Sim ☐

Factores de risco

1. Alguma vez fez o teste HIV e ao vírus da Hepatite B (HBV)?

Não ☐

Sim ☐

2. Conhecimento sobre HIV e Hepatite B:

Desconhece ☐

Já ouviu falar, mas não sabe nada a respeito ☐

Sabe alguma coisa sobre a doença ☐

3. Na residência, possui saneamento básico? (água tratada, colheita de lixo, sistema de esgoto):

Sim ☐

Não ☐ Se não, quais não possui?

Água ☐

Esgoto ☐

Lixo ☐

4. Tem animais de estimação actualmente em casa?

Não ☐

Sim ☐ Se sim, qual? Gatos ☐

Cães ☐

Outros ☐

Qual é o número destes animais no domicílio? ___________.

Os animais têm acesso ao interior de sua residência?

Não ☐

Sim ☐

5. Atualmente tem contacto, com frequência, com animais, que não os seus, em casa de parentes ou outras residências?

Não ☐

Sim ☐ Se sim, quais? Gatos ☐

Cães ☐

Outros ☐

6. Tem contacto com outros gatos que não sejam os seus?

Não ☐

Sim ☐ Se sim com que frequência?

Muito frequente ☐

Pouco frequente ☐

Raramente ☐

7. Caso tenha gatos de estimação, onde é que eles defecam?

Dentro da casa ☐

Na proximidade da casa ☐

Distante da casa ☐

8. Caso tenha gatos de estimação, qual é o seu principal alimento?

Ração ☐

Restos de comida (cozinhada) ☐

Restos de carne crua ☐

9. Sabe da existência de roedores no seu domicílio ou nas proximidades?

Não ☐

Sim ☐

10. Tem horta na sua residência?

Não ☐

Sim ☐ Se sim esta é cercada, evitando a entrada de gatos?

Não ☐

Sim ☐

11. Realiza atividades ligadas ao solo (jardinagem, agricultura, etc.)?

Não ☐

Sim ☐

12. Tem animais de criação para consumo próprio?

Não ☐

Sim ☐ Se sim, quais?

Gado ☐

Porcos ☐

Aves ☐

☐

Outros

12. Tem hábitos de consumir carne proveniente de animais abatidos em caça como pássaros, coelhos, javalis etc.

Não ☐

Sim ☐

13. Consome carne crua ou mal cozida?

Não ☐

Sim ☐ Se sim, de que animais? Gado ☐

Porco ☐

Ave ☐

Outros ☐

Se sim, com que frequência?

Muito frequente ☐

Pouco frequente ☐

Raramente ☐

14. Consome leite ou lacticínios não pasteurizados?

Não ☐

Sim ☐ Se sim, com que frequência?

Muito frequente ☐

Pouco frequente ☐

Raramente ☐

15. Lava frutas e verduras sempre antes de consumi-las?

Não ☐

Sim ☐ Se sim, com que frequência?

Muito frequente ☐

Pouco frequente ☐

Raramente ☐

16. Consome ovo cru ou mal cozido?

Não ☐

Sim ☐ Se sim, com que frequência?

Muito frequente ☐

Pouco frequente ☐

Raramente ☐

17. Consome água de poço/furo?

Não ☐

Sim ☐ Se sim, com que frequência?

Muito frequente ☐

Pouco frequente ☐

Raramente ☐

18. Recentemente fez alguma transfusão de sangue?

Não ☐

Sim ☐

19. Recentemente teve alguma picada/acidente de agulha/seringa?

Não ☐

Sim ☐
